# The upregulation of circFoxp1 influences keloid by promoting cell proliferation

**DOI:** 10.18632/aging.205215

**Published:** 2023-11-21

**Authors:** Jing Zhang, Qianyi Bao, Nan Song, Chunming Li, Jing Ma

**Affiliations:** 1 Department of Facial Plastic and Reconstructive Surgery, ENT Institute, Eye and ENT Hospital, Fudan University, Shanghai, China; 2 School of Medicine, Southeast University, Nanjing, Jiangsu, China; 3 Key Laboratory for The Genetics of Developmental and Neuropsychiatric Disorders (Ministry of Education), Bio-X Institutes, Shanghai Jiao Tong University, Shanghai, China; 4 Weigao Fenwei Health Technology Development (Shanghai) Co., Ltd., Shanghai, China

**Keywords:** keloid, circRNA, cell proliferation, oxidative stress

## Abstract

As a result of abnormal wound healing in susceptible individuals, keloids are characterized by hyperproliferation of fibroblasts and excessive deposition of the extracellular matrix (ECM). Current surgical and therapeutic modalities provide limited satisfactory results. Circular ribonucleic acids (circRNAs) play a crucial role in the pathogenesis of various fibrotic diseases, but the potential biological function and expression profile of circRNAs in keloid formation remain unknown. In this study, we explored the function of circFoxp1 on keloid formation. Methods: Quantitative reverse transcription-polymerase chain reaction (qRT-PCR) results revealed that circFoxp1 expression was higher in the keloid tissues. Furthermore, RNA-fluorescence *in situ* hybridization (RNA-FISH) and RNAscope illustrated that circFoxp1 was present in the cytoplasm. Subsequent cellular experiments demonstrated that circFoxp1 overexpression enhanced proliferation, migration, and ECM deposition. In addition, apoptosis was inhibited. Cell proliferation, inflammatory response, and oxidative phosphorylation of fibroblasts were also observed by RNA sequencing and were closely related to scar formation. The therapeutic potential of circFoxp1 was investigated by establishing keloid implantation models. *In vivo*, circFoxp1 can promote fibroblast proliferation and ECM deposition. RNA pull-down and western blot assays verified the interaction of circFoxp1 with RACK1. The present study reveals that circFoxp1 contributes to the pathological hyperplasia of keloid, which may improve inflammation and cell proliferation. Our data indicate that circFoxp1 may serve as a novel, promising therapeutic target, presenting a new avenue for understanding the underlying pathogenesis of keloid.

## INTRODUCTION

Observed only in humans, keloids are benign fibroproliferative tumors that culminate in abnormal dermal fibrosis and are characterized by excess fibroblast proliferation and extracellular matrix (ECM) (particularly collagen levels) deposition followed by abnormal wound healing [1]. Patients may experience itching, pain, restricted movement, and interference with daily activities [2]. Keloid can also be complicated by ulceration, bleeding, and infection — seriously affecting patients’ physical and mental health [3]. As the main cellular component in keloid tissue, keloid-derived fibroblasts migrate outside the typical wound region and overproduce a high level of ECM synthesis, indicating an association between continuous invasive growth and collagen production [4]. Simultaneously, a balance between proliferation and apoptosis of fibroblasts is disrupted in keloid patients, resulting in the constant hyperplasia of the keloid. Various strategies have been tested for keloid treatment, including surgery, radiotherapy, pressure, intralesional steroids, interferon, 5-fluorouracil injections, laser therapy, cryotherapy, and silicone gel sheeting [3]. Unfortunately, recurrence and spreading rates remain high, and most patients do not experience satisfactory therapeutic outcomes. Consequently, insights into the intricate molecular mechanisms underlying invasive keloid progression may be valuable for exploring novel and effective alternative strategies for treating keloid.

Circular ribonucleic acid (circRNA) is a newly described RNA molecule that forms a covalently closed continuous loop by back-splicing or lariat formation [5]. In contrast to linear RNA, circRNA is generally characterized by a high GC content, stability, diversity, and conservatism. Additionally, it plays a significant role in RNA interaction networks [6, 7], but the function of most circRNAs remains largely unknown. However, increasing evidence has demonstrated that they could serve as sponge adsorption for microRNAs (miRNAs) and RNA-binding proteins (RBPs) to modulate transcription, splicing, and translation [8–10]. Owing to advances in high-throughput sequencing technology and bioinformatics analysis, circRNAs have been found to play essential roles in various biological processes, including fate decisions, proliferation, apoptosis, migration, invasion, and differentiation. The knockout of CircPTPN12 may enhance the proliferation, migration, and aggression of keloids and decrease the apoptosis of associated keloid fibroblasts (KFBs). In terms of mechanism, the circPTPN12/MiR-21-5p/SMAD7 axis plays a vital role during keloid formation [11], and a significant increase in circPDE7B has been observed in human keloid tissues and human KFBs, accompanied by miR-661 downregulation and FGF2 upregulation. CircPDE7B directly sponged miR-661, which then governed FGF2 gene expression, suggesting a novel circPDE7B/miR-661/FGF2 pathway underlying keloid formation and possible treatment [12]. However, the mechanism of the circRNAs reported in these papers mainly focused on the role of acting as miRNA sponges. It would be helpful to gain deeper insight into the role and mechanism of circRNA in keloid formation and progression.

Based on our group’s published circRNA sequencing analysis [13] and qRT-PCR validation in expanded samples, circFoxp1 (derived from the 8–12 exon region of the *FOXP1* gene) was markedly upregulated in human keloid tissues. CircFoxp1 promotes many carcinomas and pulmonary fibrosis [14–16]. Our keloid implantation nude mouse models with circFoxp1 overexpression suggested that circFoxp1 promoted fibroblast proliferation and ECM deposition. *In vitro* experiments further indicated that circFoxp1 aggravated keloid progression by promoting fibroblast proliferation, apoptosis, inflammation response, and oxidative stress. Thereafter, our RNA pull-down experiment identified that the RNA binding protein RACK1 is a potential target of circFoxp1 and may serve as a regulator of cell proliferation and apoptosis throughout keloid formation. RACK1 can inhibit collagen synthesis within keloid fibroblasts via inhibition of the TGF-β1/Smad signaling pathway [13]. Results of our current study suggest that circFoxp1 may act as a potential regulator involved in keloid progression.

## METHODS

### Tissue samples

Keloid tissue samples were obtained from 70 patients treated for cosmetic resection at the Eye, Ear, Nose, and Throat (EENT) Hospital of Fudan University. Controls included normal tissue samples of the uninvolved skin near the keloids of 29 enrolled participants and normal skin tissue samples of 41 individuals without keloids. Written informed consent from each patient was obtained before any samples were collected or analyzed. Pathologists verified the pathological features of the samples. This research was approved by the ethical committee of the EENT Hospital of Fudan University (2020027).

### Human skin fibroblast (HSF) culture and infection

An established protocol was used to culture primary human dermal fibroblasts [17]. After separating the epidermis from tissue samples, the dermis was washed three times in phosphate-buffered saline (PBS) and cut into pieces of approximately 2 × 2 × 2 mm. To digest the tissue, about 6 mg of each tissue was digested in 5.5 mL containing 2.0 mg/mL collagenase type I (Solarbio, Beijing, China) and 2.5 mg/mL trypsin (Gibco, USA). Resulting HSFs were washed with PBS, filtered through a cellular membrane, and cultured in Dulbecco’s Modified Eagle’s Medium (DMEM, Gibco, USA) containing 15% fetal bovine serum (FBS, Gibco, USA) and 1% penicillin/streptomycin at 37°C in a 5% CO2 atmosphere. Further experiments were conducted using the third to fifth passages of HSFs. Before infection, HSFs were seeded in six-well plates at a density of 10^5^/well and incubated to 70–80% confluence. Genechem Co., Ltd. (Shanghai, China) designed and synthesized the lentiviruses that encode circFoxp1 (OE-circFoxp1) and its negative control (OE-NC). Real-time quantitative RT-PCR was conducted after 72 hours of incubation to determine the infection efficiency of circFoxp1 overexpression lentiviruses.

### Cell proliferation assay

The cell counting assay kit-8 (CCK-8, Sigma-Aldrich, USA) was used to assess the proliferation of HSFs, following the manufacturer’s protocol. HSFs were plated into 96-well plates at 3 × 10^3^/well and incubated to 40% confluence. Each well sample was then treated with CCK-8 reagent (at 0, 24, 48, and 72 hours after transfection) and incubated for 2 hours in the dark. Optional density was then measured at 450 nm with a microplate reader (BioTek Instruments, USA).

### Colony formation assay

The above HSFs were transferred into six-well plates at 5 × 10^5^/well for the colony formation assay. Following two weeks of incubation at 37°C, HSFs were fixed in 4% paraformaldehyde for 20 minutes at 25°C and stained with 0.1% crystal violet (Beyotime Biotechnology, Cat. No. C0121, Shanghai, China). Colonies were observed by digital light microscopy at 200× magnification (Olympus CKX41, U-CTR30-2, Japan). Three observers independently measured and photographed the colonies with a Canon EOS M50 digital camera (Canon, Inc., Japan).

### Transwell migration assay

The migratory capacity of HSFs was evaluated using 24-well, 8 μm pore size Transwell migration champers (Corning, USA). Briefly, 200 μL of serum-free DMEM media was used to resuspend the HSF samples to 5 × 10^4^/well before uniformly injecting them into the inner chambers. The bottom chambers were refilled with 500 μL DMEM media that contained 15% FBS as an attractant. After 24 hours, cells that migrated to the bottom chamber were fixed with 4% paraformaldehyde and stained with 0.1% crystal violet. Finally, the number of migrating cells was counted using ImageJ software (NIH, USA).

### Flow cytometric assays

The apoptosis rate of the HSFs was determined using the Annexin V-FITC/propidium iodide (PI) Apoptosis Detection Kit (KeyGen Biotech, Nanjing, China). HSFs were seeded at a density of roughly 2 × 10^5^/well on six-well plates. Following infection, the cells were isolated using 0.25% pancreatin without EDTA and rinsed thrice with cold PBS. The cells were resuspended in 500 μL binding buffer, treated with 5 μL Annexin V-FITC for 15 minutes, and then 5 μL PI for 5 minutes in the dark. Finally, flow cytometry (BD FACSCalibur, USA) was used to determine the cell apoptosis rate in each tube. To assess the impact of the intervention variables on cell apoptosis, the percentages of early (Annexin V-FITC+/PI) and late (Annexin V-FITC+/PI+) apoptotic cells in each sample were computed using FlowJo software version 8 (USA).

### Fluorescence *in situ* hybridization

The intracellular location of circFoxp1 in HSFs was identified by FISH analysis. Cy3-labeled circFoxp1 probes were designed and synthesized by Geneseed Biotech, China. The cells were rinsed with PBS, fixed in 4% formaldehyde solution for 10 minutes at 37°C, and then incubated in 0.5% Triton X-100 solution for 5 minutes at 4°C. *In situ* hybridization with circFoxp1 probes was carried out overnight in the dark. Images were captured by fluorescence microscopy at 400× magnification (TCS SP2/AOBS, Olympus, Japan).

### RNA scope assay

The RNAscope assay followed the manufacturer’s instructions (Advanced Cell Diagnostics, USA). Tissue samples were embedded in paraffin, then sectioned at 10 μm thickness, mounted on Superfrost Plus slides (Thermo Fisher Scientific, UK), and dried at 25°C overnight. The sections were then baked at 60°C for 1 hour before deparaffinization with xylene (twice for 5 minutes) and ethanol (twice for 2 minutes), then dried by baking at 60°C for 2 minutes. Then, target retrieval was carried out with sequential steps of 15 minutes at 100°C, hydrogen peroxide for 10 minutes at 25°C, and RNAscope Protease IV for 30 minutes at room temperature. In between each treatment, the samples were washed twice with distilled water. RNAScope probes (BA-Hs-FOXP1-circRNA-E12E8-Junc-C1, #1120681-C1) and positive controls were applied. The samples were incubated for 2 hours at 40°C in a HybEZ oven and then with sequential reagent steps of AMP1 (30 minutes at 40°C), AMP2 (30 minutes at 40°C), AMP3 (15 minutes at 40°C), AMP4 (30 minutes at 40°C), AMP5 (30 minutes at 40°C), AMP6 (15 minutes at 40°C), AMP7 (60 minutes at RT), and finally AMP8 (30 minutes at RT). Between the AMP incubation phases, the slides were rinsed with wash buffer (three times for 2 minutes). They were then counterstained with Gill’s hematoxylin, dried for 15 minutes at 60°C, treated with Fast Red for 10 minutes at 25°C in the dark, and mounted with Catamount permanent mounting medium (Vector Labs, USA).

### Implantation of keloid tissue into nude mice

The use of animals in this study was approved by the Animal Care and Use Committee of the EENT Hospital of Fudan University. Patients’ keloid specimens were carefully cut into small pieces (4 × 3 × 3 mm). Six-week-old male BALB/C nude mice (*n* = 10 for each group) were treated under general anesthesia to examine the therapeutic impact of circFoxp1 on keloids *in vivo* (Scheme 1). Within 3 hours of resection from patients, two tiny samples of keloid tissue were implanted into each of the thirty nude mice (one on the right lower back, and one on the left lower back). All transplanted sites were at least 3 cm apart to avoid treatment diffusion and cross-treatment impact. After wound healing (about two weeks), each mouse was randomly assigned to one of three treatment groups (control, OE-NC, and OE-Foxp1). The keloid grafts were removed from the nude mice after two weeks and six weeks after the operation. Weight and graft volumes were recorded. The following histological evaluation and western blot analyses were performed on the keloid grafts.

### Immunohistochemistry (IHC)

The transplanted keloid tissues from nude mice were sectioned at 4 mm, fixed in methanal, and then embedded in paraffin. Sections of 5 μm thickness were mounted on ten slides and dehydrated in ethanol solutions after being deparaffinized in xylene. The sections were immersed in TBST buffer solution, placed in a wet box, covered with the enzyme repair solution (1:500 preparation), and incubated in a warm box at 37°C for 45 minutes. After incubation, the sections were immersed in TBST three times for 5 minutes and blocked in goat serum for 30 minutes to prevent non-specific binding. They were then incubated at 4°C overnight with primary antibodies (1:400 dilution; Boster, BA0325). Next, the secondary antibody (1:2000 dilution; Abcam ab205718) was added, and incubated in a warm box at 37°C for 45 minutes. The sections were stained with diaminobenzidine to detect positive signals, and nuclei were counterstained with hematoxylin (Damjanovic et al., 2020).

### qPCR

RNA was reverse transcribed with HiScript II Q RT SuperMix for qPCR (+gDNA wiper) (Vazyme, Nanjing, China), and amplified with qPCR SYBR Green Master Mix (Vazyme, Nanjing, China). GAPDH was used to standardize the amounts of the circRNA and mRNA. The Supplementary Tables 1 and 2 includes a list of oligonucleotide sequences.

### Western blot analysis

An equal amount of total protein lysates (20 μg) was separated by 8% SDS-PAGE and transferred onto a PolyVinyliDene Fluoride (PVDF) membrane. The membranes were blocked for nonspecific binding and incubated with the primary antibody a-SMA (1:3000, Abcam, ab5694) and Col1a1 (1:1000, CST, 72026) overnight at 4°C. Incubation with the secondary antibody was then conducted for 1 hour at room temperature. Signals were developed using the ChemiDoc XRS system (Bio-Rad, USA).

### RNA-seq analysis

Total RNA samples were extracted with Trizol (Invitrogen, USA). RNA integrity was analyzed with an Agilent 2100 Bioanalyzer (Agilent Technologies, USA). Acceptance criteria for total RNA samples were as follows: RNA integrity number (RIN) ≥7.0 and a 28 S:18 S ratio ≥1.5. Sequencing libraries were generated and sequenced using LC-Bio Technology CO., Ltd., (Hangzhou, China).

### Reactive oxygen species measurement

Reactive oxygen species (ROS) was measured with the CellROX Orange Reagent (C10448; Invitrogen, USA) according to the manufacturer’s instructions. Briefly, cells were probed with 5 μM CellROX Orange at 37°C for 30 minutes, washed with ice-cold PBS, and counterstained with DAPI (10236276001; Roche Diagnostics GmbH) for 10 minutes to label the nuclei. The cells were mounted and examined with a Leica TCS SP8 laser scanning confocal microscope. ImageJ was used to measure the intensity of the red fluorescence.

### RNA pulldown and mass spectrometry analysis

For RNA pull-down assay, 1 × 10^7^ cells were washed in ice-cold PBS, lysed in 400 μL Dilution buffer (Thermo Fisher Scientific) supplemented with a cocktail of proteinase inhibitors, phosphatase inhibitors, and RNase inhibitor (Invitrogen, USA), and then incubated with 3 μg biotinylated RNA probes against circFoxp1 back splice junction region (sense) or corresponding complementary probes (antisense) for 2 hours at room temperature. A total of 50 μL of washed Pierce Nucleic-Acid Compatible Streptavidin Magnetic Beads (Invitrogen, USA) were added to each binding reaction and further incubated for another hour at room temperature. The beads were washed briefly with a wash buffer five times. Following washing, the retrieved protein was detected by Western blot or mass spectrometry analysis. Probe sequences are listed in Supplementary Tables 1 and 2.

### Statistics

The results are displayed as the mean ± standard deviation (SD). Prism software (GraphPad Software 8) was used for the statistical analyses, including analysis of variance and Student’s *t*-test to compare the two experimental groups. It was agreed that a probability of 0.05 or less was statistically significant.

## RESULTS

### CircFoxp1 expression is upregulated in keloid tissues

**Figure 1:**
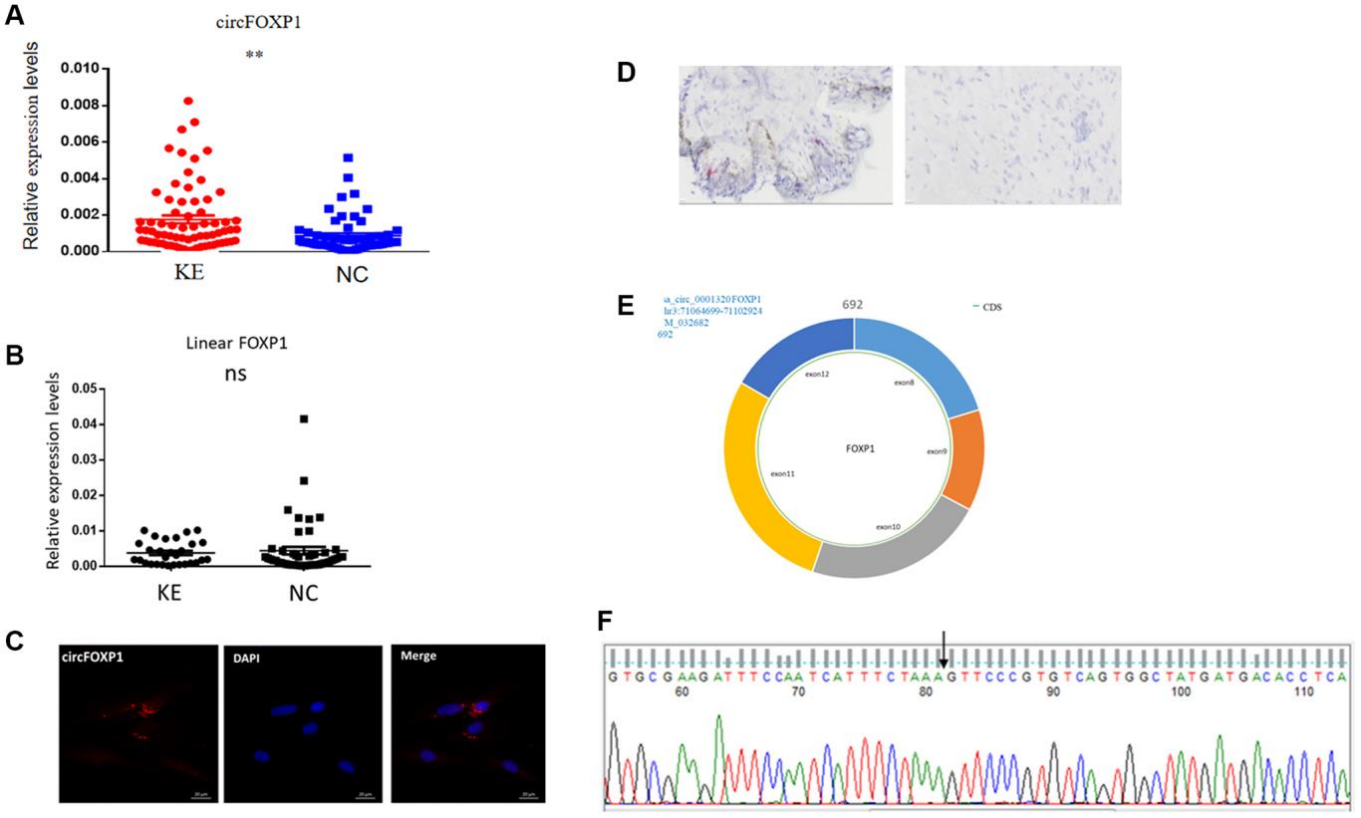
**Expression levels and correlation analysis of has-circFoxp1 and linear Foxp1 mRNA in keloid tissues.** (**A**) qRT-PCR analysis of relative RNA levels of circFoxp1 in keloid tissues and normal skin. (**B**) qRT-PCR analysis of relative RNA levels of Foxp1 in keloid tissues and normal skin. (**C**) FISH assay indicting the cellular location of circFoxp1 (red) in the HSFs (magnification 400× and scale bar 20 μm). Probe sequence: TGACACGGGAACTTTAGAAATGAT. (**D**) RNAscope assay indicating the cellular location of circFoxp1 (red) in Keloid tissues (magnification 100× and scale bar 100 μm). Most of the expression of circFoxp1 was located in the cytoplasm. (**E**) Map of circFoxp1 derived from the 8-12 exon region of the FOXP1 gene. (**F**) Sanger sequencing results indicating the cyclization site of circFoxp1 (arrow). ^**^p < 0.001.

A previous high-throughput sequencing study showed that a higher level of circFoxp1 occurred in keloid tissues than in normal skin tissues [17]. The relative expression of circFoxp1 was first detected by qPCR in keloid and normal skin tissues. In agreement with the high-throughput sequencing results, circFoxp1 was significantly upregulated in the keloid tissues, whereas the *Foxp1* expression level did not change (Figure 1A, 1B). In addition, circFoxp1 was generated from the Foxp1 gene located on chromosome 3p14.1. It resulted from the head-to-tail splicing of exon 8 and exon 12 (CircBase ID: hsa_circ-0001320, splicing sequence length: 692 nt, 3:71064700|71102924) (Figure 1E). According to subcellular localization studies using FISH and RNAscope (in HSFs and keloid tissues, respectively), circFoxp1 was primarily located in the cytoplasm, indicating that circFoxp1 might function there (Figure 1C, 1D). Sanger sequencing results showed that circFoxp1 had a cyclization site, confirmed as circRNA (Figure 1F). CircFoxp1 has been identified as a true circular RNA. These data demonstrate that circFoxp1 is closely correlated with the progression of human keloid.

### CircFoxp1 regulated HSF proliferation, migration, and apoptosis

**Figure 2:**
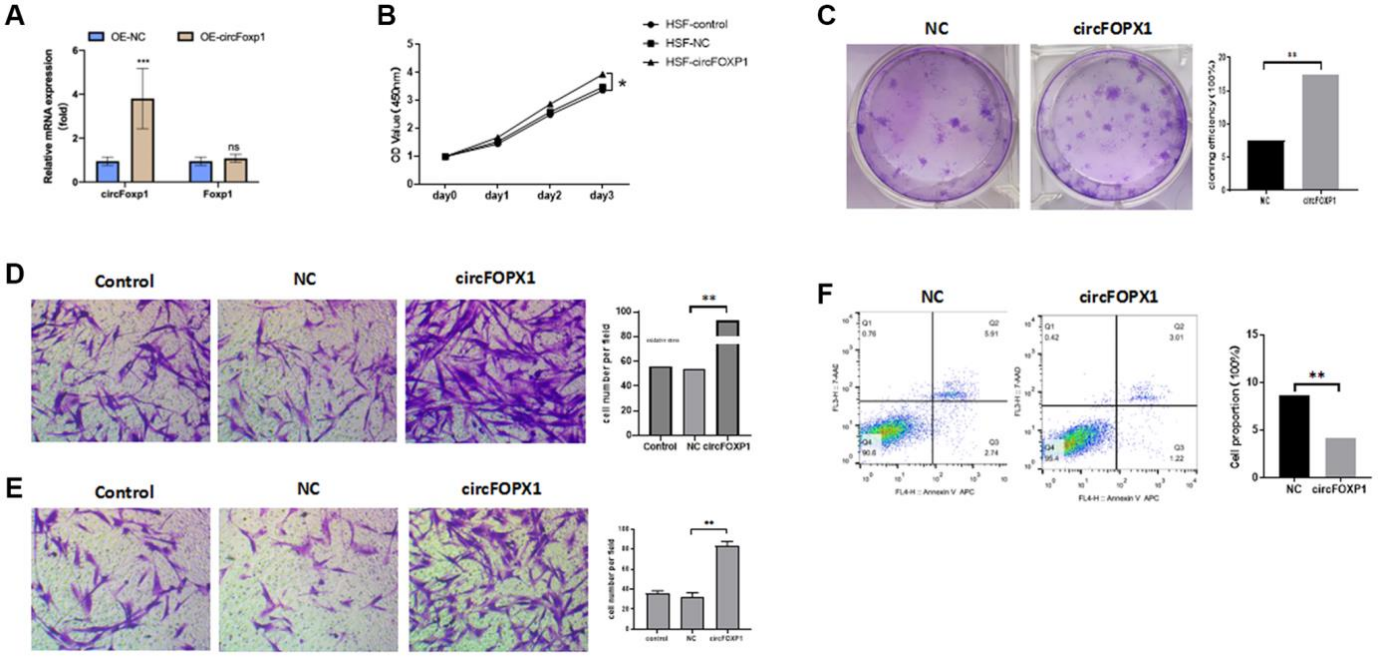
**circFoxp1 regulates HSF proliferation, migration, apoptosis, and ECM deposition *in vitro*.** (**A**) qRT-PCR analysis of overexpression efficiency of circFoxp1 in HSFs transfected with OE-NC or OE-Foxp1 lentivirus. (**B**) CCK-8 assay of the proliferation ability in HKFs transfected with OE-NC or OE-circFoxp1. (**C**) Colony formation assays for HSF cells with circFoxp1 overexpression. (**D**) Transwell assays showing overexpression of circFoxp1 obviously enhanced cell migration in HSF compared with OE-NC cells. (**E**) Transwell assays showing overexpression of circFoxp1 obviously enhanced cell invasion in HSF compared with OE-NC cells. (**F**) Cell apoptosis analyses via flow cytometric assays in OE-circFoxp1-transfected HSF cells. *n* = 3 biologically independent samples. Data shown are mean ± SD, ^*^p < 0.05, ^**^p < 0.001.

**Figure 3:**
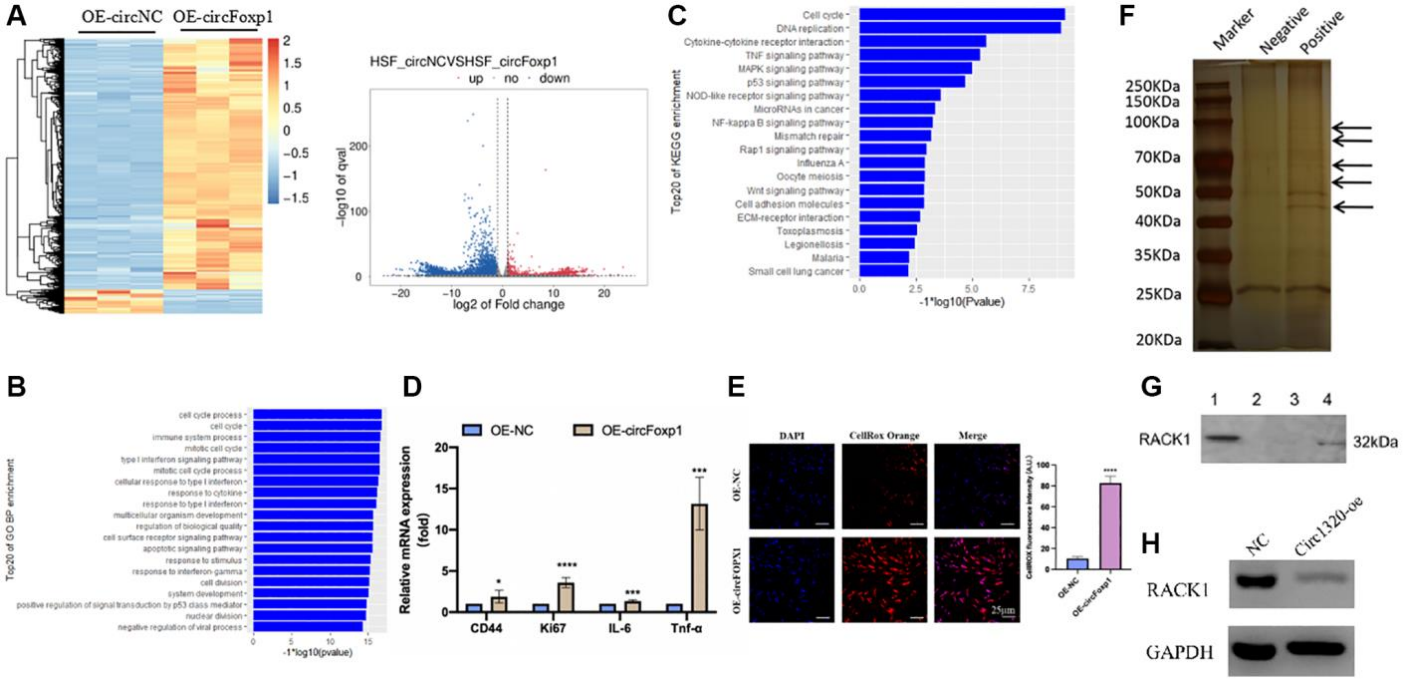
**Gene expression profiling reveals an increase in genes associated with inflammation and RNA pull-down shows circFoxp1 interact with RACK1.** (**A**) Heatmap and volcano plot showed the differentially expressed RNAs in paired samples of HSFs by RNA-seq analysis. Fold change > 1 or <−1.0, *P* value < 0.05. (**B**) GO annotations of the genes of upregulated expressed RNAs. The bar plot presented the enrichment scores (−loge [*p* value]) of the top 20 significantly enriched GO terms in biological processes, cellular components and molecular functions. (**C**) Bulb map of KEGG analysis for the genes of upregulated expressed RNAs. Rich factor represented the enrichment degree of differentially expressed genes. Y axis showed the name of enriched pathways. The area of each node represented the number of the enriched host genes of differentially expressed RNAs. The p-value was represented by a color scale. (**D**) Quantitative RT-PCR to quantify fibroblast migration marker (CD44), proliferation marker (Ki67) and inflammation cells marker (IL-6 and TNF-α) circFoxp1 overexpressed HSF cells. ^*^P < 0.05, ^***^P < 0.001. (**E**) ROS activity was determined by confocal fluorescence microscopy using CellROXTM Deep Orange Staining. Scale bar: 25 μm. ^****^P < 0.0001. (**F**) RNA pulls down revealed several proteins interact with circFoxp1. (**G**) Western blot confirmed ROCK1 presented in RNA pulldown liquid. (**H**) ROCK1 reduced in the OE-circFoxp1 group as compared to OE-Ctr in HSFs.

To further investigate the circFoxp1 impact *in vitro*, circFoxp1 overexpression lentivirus (OE-circFoxp1) and control lentivirus (OE-NC) plasmids were designed and synthesized to specifically upregulate circFoxp1 in HSFs without affecting the level of Foxp1 mRNA. A high level of circFoxp1 overexpression efficiency was achieved (Figure 2A). To test cell viability and proliferation, a CCK-8 assay and colony formation assay were performed. Based on the CCK-8 results, OE-circFoxp1 significantly enhanced the proliferation of HSFs, particularly 72 hours after virus infection (Figure 2B). Similarly, in the colony-forming assay, the OE-circFoxp1 group formed more colonies than the OE-NC group (Figure 2C). Supporting this, Transwell assays showed that overexpression of circFoxp1 can lead to significant increases in cell motility and invasion (Figure 2D, 2E). As shown in Figure 2F, the upregulation of circFoxp1 significantly decreased the apoptotic ratio of the HSFs. We observed that the ratio of viable cells drastically increased, while apoptosis and necrosis decreased significantly. This was consistent with the results of the CCK-8 and colony formation assays. Both gene ontology (GO) and Kyoto Encyclopedia of Genes and Genomes (KEGG) analyses of RNA-seq revealed that cell cycle and DNA replication are the top two pathways that circFoxp1 overexpression genes were enriched in (Figure 3B, 3C). These genes were all associated with cell proliferation. In addition, qRT-PCR results of circFoxp1 overexpressed HSF cells also confirmed that circFoxp1 promotes the expression of genes related to cell proliferation (Figure 3D).

### CircFoxp1 upregulation promotes the growth and ECM deposition of keloid *in vivo*

**Figure 4:**
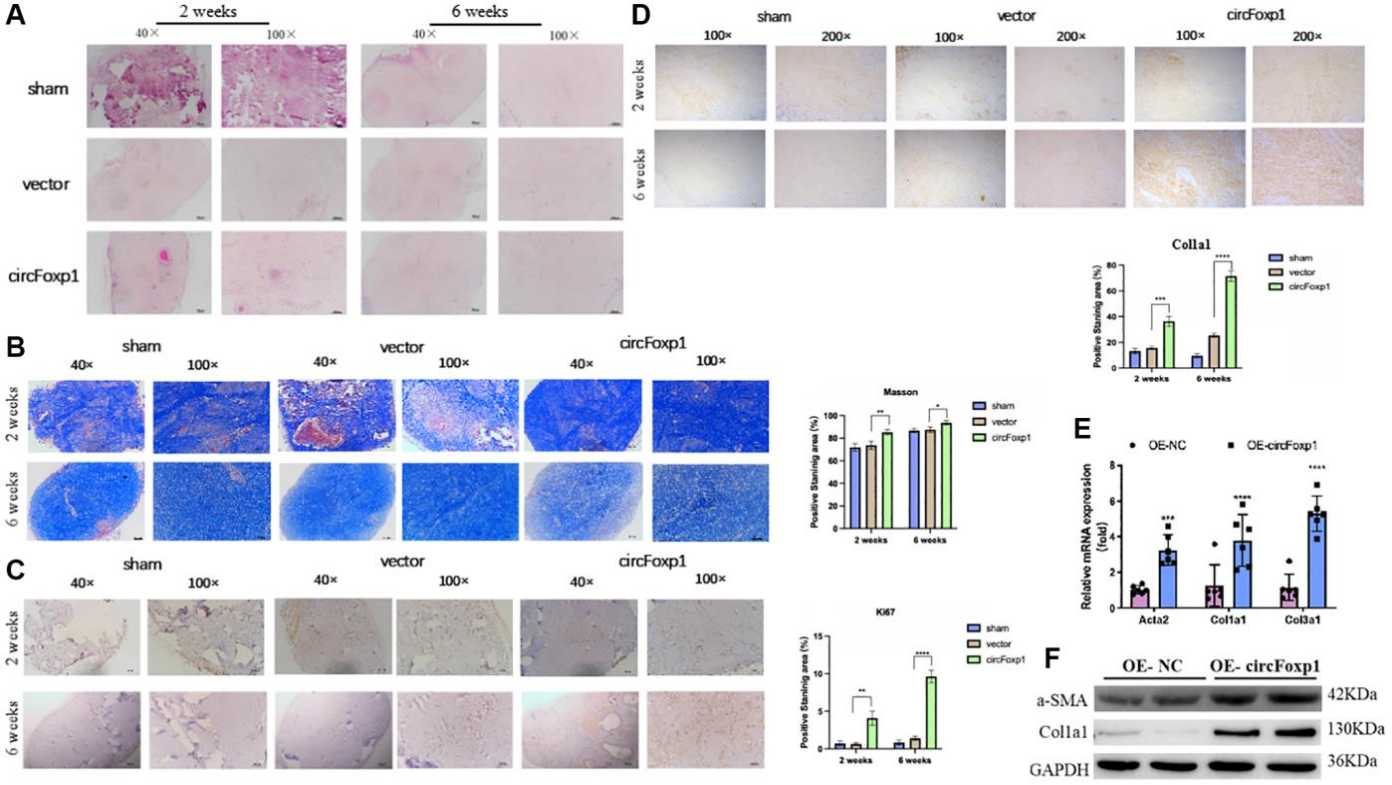
**Overexpression of circFoxp1 enhances the growth of keloid and ECM deposition *in vivo*.** (**A**) Representative images of HE staining and (**B**) Masson staining of keloid nodules in different intervention groups. (magnification, 100× and magnification, 200×). (**C**) Relative expression level of Ki67 and (**D**) Col1a1 observed in keloid grafts by IHC. (magnification, 200× and magnification, 400×). ^*^P < 0.05, ^**^P < 0.01, ^***^P < 0.001, ^****^P < 0.0001. (**E**) Quantitative RT-PCR (*n* = 6) showing transcript expression of related markers of ECM upregulated compared with the OE-NC of HSFs. (**F**) Western blot (*n* = 3) showing protein expression of related markers of ECM synthesis upregulated compared with the OE-NC of HSFs.

To further elucidate the contribution of circFoxp1 to the growth and ECM deposition in keloid, fresh human keloid tissues were implanted into nude mice to generate keloid models, which were then treated with PBS, a control vector, or circFoxp1 overexpressing lentiviruses. To determine the characteristics of subcutaneous transplanted keloid tissues, they were stained with hematoxylin-eosin (HE) and Masson’s trichrome stain. Microscopic imaging showed that the refractory homogeneous lamellar collagen fibers of the keloid tissues in the circFoxp1 group became thicker, highly dense, and tightly arranged compared with the sham and control groups (Figure 4A, 4B). In addition, IHC analysis revealed that the protein expression of ki67 and type I collagen in the circFoxp1 group was significantly higher than in the control tissues (Figure 4C, 4D). To better understand the function of circFoxp1 in promoting the pathological hyperplasia of keloid, the presence of ECM components, including α-SMA, collagen I, and collagen III, was detected by qRT-PCR. As expected, these results revealed that the expressions of type I and III collagen (and α-SMA) were markedly upregulated upon circFoxp1 infection in HSFs (Figure 4E). Furthermore, western blotting analysis demonstrated increased ECM synthesis (Figure 4F).

These findings demonstrate that circFoxp1 can aggravate the pathological phenotype of keloid and that circFoxp1 is a potential therapeutic target.

### CircFoxp1 upregulation exacerbates the inflammatory response and inhibits RACK1 expression

To further clarify the role of circFoxp1 in fibroblast and scar formation, RNA-seq analysis of circFoxp1 overexpressed HSF cells was performed (Figure 3A). Consistent with the cell function results, GO and KEGG analyses revealed that circFoxp1 overexpression increased genes associated with the cell cycle, inflammation, and migration. Notably, the circFoxp1 overexpressed cells showed a dramatic increase in the pathways related to the inflammatory response (Figure 3B, 3C). Subsequently, we further verified proliferation, migration, and inflammation-related markers using qRT-PCR. The results showed that overexpression of circFoxp1 can aggravate pathological changes related to scar formation (Figure 3D). Oxidative stress can induce an inflammatory response. Therefore, we predict that circFoxp1 overexpression promotes HSF proliferation and exacerbates the cellular oxidative stress response, further aggravating inflammation *in vivo*, affecting wound repair, and leading to scar formation. ROS are essential mediators in various pathways, including apoptosis and autophagy. Thus, we investigated the role of ROS generation in circFoxp1 overexpressed HSF cells. As expected, overexpressed circFoxp1 drastically increased ROS levels in HSF cells (Figure 3E). To enquire how circFoxp1 activation impresses keloid progression, we explored RNA binding proteins that interact with circFoxp1 to exert functions. Firstly, an RNA pull-down assay was examined to identify the proteins combined with it. In the pull-down assay, the precipitates were separated by SDS-PAGE and visualized by silver staining. The sense-specific bands (black arrow) were excised for mass spectrometry (Figure 3F). As one of the RNA-binding proteins, RACK1 is an omnipresent and multifunctional protein located in the cytoplasm. We then confirmed the mass-spectrometry results by Western blot assay (Figure 3G); simultaneously, the amount of RACK1 going down in HSFs overexpressed circFoxp1 was performed (Figure 3H).

## DISCUSSION

Keloid is a common dermal fibroproliferative disorder characterized by aggressive proliferation of fibroblasts, enhanced synthesis of collagen, and the accumulation of myofibroblasts. The etiology of keloid remains undeciphered but may be closely involved in a complex interplay of genetic and environmental factors [1, 18]. Although several common therapeutic approaches have been applied for keloid prevention and treatment (surgical excision combined with local radiotherapy, steroid injections, and compression therapy), they are largely ineffective and often relapse [19]. Epigenetic modifications, including DNA methylations, histone modifications, and ncRNA regulations, are emerging, challenging research fields illuminating keloid investigations’ molecular pathogenesis. Following technical advances in sequencing, intensive research of the transcriptome-wide range has indicated that ncRNAs are crucial in coordinating function and gene transcription in keloid pathogenesis [20]. Based on a previous study, circCOL5A1 acted as a ceRNA by absorption of miR-7-5p to release Epac1 and regulate human keloid fibroblast proliferation, migration, apoptosis, and ECM deposition. Furthermore, si-circCOL5A1 resulted in suppressing the Epac1-induced PI3K/AKT signaling pathway to partially reverse the pathological phenotype of hyperproliferation and invasive growth, suggesting that circCOL5A1 was involved in coordinating collagen hyperplasia and fibroblast proliferation [21]. However, the study of keloid-associated circular RNAs is still limited, and the functions of most circular RNAs remain unclear. The high-throughput sequencing results presented in this study first confirmed that the expression of circFoxp1 was upregulated in keloid tissues, as compared with normal skin tissues. Furthermore, circFoxp1 may take part in the occurrence and progression of keloid.

In this study, significant circFoxp1 upregulation was observed in keloid patients by qPCR, consistent with the previously published results of high-throughput circRNA sequencing [17]. CircFoxp1 has been reported to promote the proliferation and differentiation of BMSCs by epigenetically repressing PTEN and, therefore, activating the AKT pathway [22]. In addition, circFOXP1 could promote cell proliferation and repressed cell apoptosis in lung adenocarcinoma [23]. Similarly, we found that circFoxp1, derived from the 8-12 exon region of the *FOXP1* gene, acts as a fibrotic promoter *in vitro* to accelerate HSF proliferation, migration, and ECM deposition, as well as cell apoptosis inhibition. In addition, nude mice were implanted with keloid tissue to confirm the role of circFoxp1 in promoting ECM deposition *in vivo*. These results suggested that circFoxp1 was upregulated in human keloid tissues, stimulates collagen hyperplasia, and could be considered a potential target for fibrosis curing.

Inflammatory conditions appear to play a relevant role in the pathogenesis of various fibrotic diseases. Considering these previous reports, the fibrotic and inflammatory status of keloid may be associated with ROS production. Using cellular RNA-seq analysis, we found that overexpression of circFoxp1 in fibroblasts may promote cell proliferation and exacerbate the inflammation response, a critical pathological process in keloid formation. Moreover, inflammation-related signaling pathways are significantly enriched in circFoxp1-overexpressed fibroblasts, indicating that circFoxp1 may regulate the inflammatory response through oxidative stress and correlate with keloid formation. Additionally, our ROS fluorescence staining supported this. These results suggest that circFoxp1 governs the inflammatory response by promoting the production of cellular ROS, which may promote collagen hyperplasia and correlate with keloid formation.

CircRNA in the cytoplasm could serve as adsorption sites for RNA-binding proteins (RBPs) to perform a specific function. Our RNA pull-down assay demonstrated that circFOXP1 could interact with RACK1 protein, and circFOXP1 overexpression was accompanied by ROCK1 decline. As revealed in a previous study, reducing RACK1 could promote keloid formation [13].

We would like to raise some limitations associated with this study that we wish to resolve in the future. First, we examined the effect of circFoxp1 overexpression in HSFs without considering whether knocking down circFoxp1 would lead to the same conclusion. Second, we have not yet investigated the impact of circFoxp1 on keloid formation under pathological conditions, which could provide convincing evidence that circFoxp1 could be a therapeutic target. Third, we should deeply investigate the mechanism of circFoxp1 in improving fibroblast proliferation and inflammation response.

## CONCLUSION

In conclusion, we found that circFoxp1 was significantly upregulated in the keloid. Our research provides the first comprehensive evidence that circFoxp1 regulates HSF proliferation, migration, apoptosis, and ECM deposition through cellular inflammatory responses and oxidative stress. Consequently, our findings elucidate the pivotal role of circFoxp1 in the etiology and pathogenesis of keloid and may shed novel light on diagnostic and therapeutic strategies for keloid.

## Supplementary Materials

Supplementary Tables
