# Supplementary material for: The upregulation of circFoxp1 influences keloid by promoting cell proliferation
Source: Aging (Albany NY). 2023 Nov 21;15(22):12998–3009. doi: 10.18632/aging.205215 (PMC10713398; doi:10.18632/aging.205215)
Supplement: Supplementary Tables [file aging-15-205215-s001.pdf]

SUPPLEMENTARY TABLES

Supplementary Table 1. Primer used for QPCR analysis.

Name	Forward primer (5′->3′)	Reverse primer (5′->3′)
hsa_circ_0001320	TGAGGAGCACCCCCATAGC	CATCATAGCCACTGACACGG
FOXP1	AAGTTTGTCCCATGAGGAGC	CTGGAGGATCTGCTGCATTT
IL-6	ACTCACCTCTTCAGAACGAATTG	CCATCTTTGGAAGGTTCAGGTTG
TNF-a	CCTCTCTCTAATCAGCCCTCTG	GAGGACCTGGGAGTAGATGAG
Col3a1	TGCTGCTGGTACTCCTGGTCTG	ACCTGGACCGCCTGGTTCAC
Acta2	GTGTTGCCCCTGAAGAGCAT	GCTGGGACATTGAAAGTCTCA
Col1a1	GAGGGCCAAGACGAAGACATC	CAGATCACGTCATCGCACAAC
CD44	CTGCCGCTTTGCAGGTGTA	CATTGTGGGCAAGGTGCTATT
Ki67	ACGCCTGGTTACTATCAAAAGG	CAGACCCATTTACTTGTGTTGGA
GAPDH	TCGACAGTCAGCCGCATCTTCTTT	ACCAAATCCGTTGACTCCGACCTT

Supplementary Table 2. Primer used for RNA pulldown.

Name	Positive-probe-biotin	Negative-probe-biotin
hsa_circ_0001320	ACACGGGAACTTTAGAAATG	CATTTCTAAAGTTCCCGTGT
